# Reproducibility of in-vivo diffusion tensor cardiovascular magnetic resonance in hypertrophic cardiomyopathy

**DOI:** 10.1186/1532-429X-14-86

**Published:** 2012-12-24

**Authors:** Laura-Ann McGill, Tevfik F Ismail, Sonia Nielles-Vallespin, Pedro Ferreira, Andrew D Scott, Michael Roughton, Philip J Kilner, S Yen Ho, Karen P McCarthy, Peter D Gatehouse, Ranil de Silva, Peter Speier, Thorsten Feiweier, Choukkri Mekkaoui, David E Sosnovik, Sanjay K Prasad, David N Firmin, Dudley J Pennell

**Affiliations:** 1 Cardiovascular Magnetic Resonance Unit, Royal Brompton Hospital, Sydney Street, London SW3 6NP, United Kingdom; 2 National Heart and Lung Institute, Imperial College, London, UK; 3 National Heart Lung and Blood Institute (NHLBI), National Institutes of Health (NIH), DHHS, Bethesda, MD, USA; 4 MR R&D, Siemens AG Medical Solutions, Erlangen, Germany; 5 Martinos Center for Biomedical Imaging, Massachusetts General Hospital, Harvard Medical School, Boston, Massachusetts, USA

**Keywords:** Hypertrophic cardiomyopathy, Diffusion tensor imaging, Diffusion weighted imaging, Cardiovascular magnetic resonance, Disarray

## Abstract

**Background:**

Myocardial disarray is an important histological feature of hypertrophic cardiomyopathy (HCM) which has been studied post-mortem, but its in-vivo prevalence and extent is unknown. Cardiac Diffusion Tensor Imaging (cDTI) provides information on mean intravoxel myocyte orientation and potentially myocardial disarray. Recent technical advances have improved in-vivo cDTI, and the aim of this study was to assess the interstudy reproducibility of quantitative in-vivo cDTI in patients with HCM.

**Methods and results:**

A stimulated-echo single-shot-EPI sequence with zonal excitation and parallel imaging was implemented. Ten patients with HCM were each scanned on 2 different days. For each scan 3 short axis mid-ventricular slices were acquired with cDTI at end systole. Fractional anisotropy (FA), mean diffusivity (MD), and helix angle (HA) maps were created using a cDTI post-processing platform developed in-house. The mean ± SD global FA was 0.613 ± 0.044, MD was 0.750 ± 0.154 × 10^-3^ mm^2^/s and HA was epicardium −34.3 ± 7.6°, mesocardium 3.5 ± 6.9° and endocardium 38.9 ± 8.1°. Comparison of initial and repeat studies showed global interstudy reproducibility for FA (SD = ± 0.045, Coefficient of Variation (CoV) = 7.2%), MD (SD = ± 0.135 × 10^-3^ mm^2^/s, CoV = 18.6%) and HA (epicardium SD = ± 4.8°; mesocardium SD = ± 3.4°; endocardium SD = ± 2.9°). Reproducibility of FA was superior to MD (p = 0.003). Global MD was significantly higher in the septum than the reference lateral wall (0.784 ± 0.188 vs 0.750 ± 0.154 x10^-3^ mm^2^/s, p < 0.001). Septal HA was significantly lower than the reference lateral wall in all 3 transmural layers (from −8.3° to −10.4°, all p < 0.001).

**Conclusions:**

To the best of our knowledge, this is the first study to assess the interstudy reproducibility of DTI in the human HCM heart in-vivo and the largest cDTI study in HCM to date. Our results show good reproducibility of FA, MD and HA which indicates that current technology yields robust in-vivo measurements that have potential clinical value. The interpretation of regional differences in the septum requires further investigation.

## Background

Hypertrophic cardiomyopathy (HCM) is the most common inherited cardiomyopathy and affects 1 in 500 of the general population [1,2]. The majority of patients experience a benign clinical course, however, a small but significant minority experience major adverse sequelae, including sudden cardiac death and heart failure mortality [2,3]. Histologically, HCM manifests as fibrosis, microvascular abnormalities, myocyte hypertrophy and myocyte disarray. Disarray may play a key role the in the genesis of the substrate responsible for malignant ventricular arrhythmias and myocardial contractile dysfunction, [4] but to date, insights into the extent and spatial distribution of myocyte disarray have been derived largely from small post-mortem series examining hearts obtained after sudden cardiac death [5-8] These studies have considerable selection bias and as a result, the in-vivo prevalence and functional consequences of disarray in HCM remain unknown. Furthermore, the diagnostic utility of disarray in HCM is unclear as the phenomenon has also been described in a variety of congenital and acquired myocardial diseases [9,10]. These include other conditions associated with left ventricular hypertrophy, such as aortic stenosis and hypertensive heart disease, [11] the latter frequently posing a challenge in the differential diagnosis of HCM [12].

Cardiovascular magnetic resonance (CMR) is assuming an increasingly important role in the diagnosis and evaluation of HCM [13,14]. It allows the accurate assessment of hypertrophy; [15] the detection and quantification of fibrosis through late gadolinium enhancement (LGE) imaging and T1-relaxometry; [16,17] and the identification of microvascular dysfunction through first-pass perfusion imaging [18]. The assessment of disarray however has proved more challenging. The emerging CMR-based technique of cardiovascular diffusion tensor imaging (cDTI) may afford this opportunity [19] and could have diagnostic utility in the differential diagnosis of left ventricular hypertrophy. This technology has been successfully applied to assess nerve fibre architecture in the central nervous system. The orientation of nerve tracts is inferred by assessing the diffusivity of water molecules which preferentially occurs parallel to the nerve fibres [20]. However, the in-vivo application of such techniques to the myocardial architecture has been limited by the challenge of resolving the molecular diffusion of water molecules in the presence of bulk motion of the heart due to cardiac contraction and respiratory motion [21,22]. Studies in animal models have suggested that this technique may give useful insights into disarray in HCM, [23,24] but to date there is only one previous in-vivo human pilot study of 5 HCM patients [25]. We previously described the development of a novel pulse sequence for cDTI which draws on technical improvements and 3 T imaging in normal subjects [22]. In the current study, we applied this novel technique to quantify the reproducibility of cDTI in HCM for Fractional Anisotropy (FA), Mean Diffusivity (MD), and Helix Angle (HA) maps to determine its robustness for potential clinical use.

## Methods

### Study population

We prospectively enrolled 10 patients with HCM referred for CMR at Royal Brompton Hospital. HCM was diagnosed in accordance with standard clinical guidelines [12]. Patients were excluded if they had atrial fibrillation, significant comorbidity, or contraindications to CMR and gadolinium-based contrast agents. The study was approved by the National Research Ethics Service and was conducted in accordance with the principles set out in the declaration of Helsinki, with written informed consent obtained from all patients.

### Image acquisition

Images were acquired using a clinical 3.0 T scanner (Magnetom Skyra, Siemens AG Healthcare Sector, Erlangen, Germany) with an anterior 18 element matrix coil and 8–12 elements of a matrix spine coil. The 10 patients were each scanned on two separate days (subsequently referred to as initial and repeat study) in order to assess interstudy reproducibility.

After the localization steps to determine the short axis of the left ventricle (LV), a retro-gated cine sequence with a temporal resolution of 40 ms, acquired in a mid-ventricular short axis plane, was used to find the timing and duration of the subject-specific end-systolic pause. Localized first and second-order shimming and frequency adjustment were performed with an adjustment box positioned to cover the extent of the left ventricle in the imaged slices. cDTI was performed in the end-systolic pause in the mid left ventricle using a diffusion-weighted stimulated echo acquisition mode (DW-STEAM) single-shot echo-planar imaging (EPI) sequence gated to every other cardiac cycle [22]. Zonal-excitation was used to reduce the field of view in the phase encode direction and therefore, the echo train length [26]. The following sequence parameters were used: b = 0 s/mm^2^ plus 6 diffusion encoding directions, fat saturation, TR = 2 RR intervals = 2000 ms (assuming a heart rate of 60 beats per minute), TE = 23 ms, BW = 2442 Hz/pixel, GRAPPA parallel imaging acceleration factor of 2, [27] field of view = 360 × 123–157 mm^2^, slice thickness 8 mm, EPI echo train length = 22–28 readouts depending on field of view, EPI readout duration = 11–14 ms, in-plane spatial resolution = 2.7 × 2.7 mm^2^ interpolated to 1.35 × 1.35 mm^2^, 3 slices, 4 mm slice gap. Typically 10 image repetitions were obtained for each slice and each diffusion direction before averaging to improve signal to noise ratio (SNR). When motion artefacts were present due to inadequate breath holding and ectopy, up to 13 repetitions were acquired and the order that the diffusion directions were acquired in was rotated to avoid losing data from the same direction at the end of each breath hold. For diffusion encoding, the maximum available on axis gradient strength of 45 mT/m was used with a trapezoidal gradient pulse duration of 10 ms, leading to a diffusion sensitivity of b = 350 s/mm^2^. Breathold duration was for 18 cardiac cycles, which was typically 18 s. The typical duration of the patient scan was 45 minutes.

In order to provide functional and morphological data, breath-hold CMR was performed. Retrospectively gated balanced steady-state free-precession cine sequences were acquired in three long-axis planes, followed by a contiguous stack of short axis slices from the atrioventricular ring to the apex [28].

### Diffusion tensor analysis

A cDTI post-processing software tool was custom-built in house using MATLAB (Mathworks, MA, USA). It included an interface to reject images corrupted by artefacts based on a visual analysis and an automatic cross-correlation algorithm to perform a rigid co-registration of the remaining images before averaging (translation, no rotation) [29]. A rank 2 diffusion tensor was generated for each voxel using the signal intensity data from the six diffusion-weighted images and the reference image where b = 0 as described in Kingsley 2006 [30]. The eigensystem (eigenvalues and eigenvectors) was then calculated for each tensor. Due to noise, high local anisotropy, misregistration, and or artefacts, a small percentage of the measured eigenvalues are negative, violating the assumption of a positive definite diffusion tensor. These were set to the mean of the corresponding non-negative eigenvalues in neighbouring voxels. Two quantitative diffusion parameter maps were calculated from the eigensystems: Fractional Anisotropy (FA) and Mean Diffusivity (MD) [31]. FA is an index of the degree of deviation of the observed diffusion from isotropic, and MD is the first moment of the diffusion tensor, which is the average diffusivity.

The principal eigenvector of the diffusion tensor was taken to represent mean intravoxel myocyte orientation. The Helix Angle (HA) was then calculated as the angle between the short axis plane and the projection of the myocyte vector onto a plane tangential to the local endocardium [32]. Left-handed epicardial helix orientations were assigned a negative angle and right handed endocardial helix orientations a positive helix angle, as originally described by Streeter, [33,34] with zero angle being aligned with the local circumferential direction.

For quantitative analysis, the myocardium was divided into 4 segments for each slice and the same segments in each slice were grouped into the conventional LV walls (anterior, septal, inferior, lateral) [35]. Care was taken to exclude papillary muscle regions. HA analysis further segmented the myocardium transmurally into endocardial, mesocardial and epicardial layers. This was performed by dividing the local myocardium into three equal thickness layers. All cDTI analysis was performed by a single observer blinded to clinical data. 3D visualisation of the tensor with superquadric glyphs [36] was implemented using Python and Paraview (Kitware, NM, USA) software. The post-processing and analysis time was approximately 3 hours.

### Image analysis

Ventricular volumes, function, mass, and ejection fraction for all patients were measured for the LV using a semi-automated threshold-based technique (CMRtools, Cardiovascular Imaging Solutions, London). All volume and mass measurements were indexed to body surface area calculated using the Du Bois method [37,38]. Late gadolinium enhancement (LGE) imaging was analysed to assess fibrosis, and recorded as present/not present with its location, and in addition the extent of LGE was quantified using CMR42 software with a full-width half-maximum threshold and expressed as percent of LV mass.

### Statistical analysis

MD and FA values were analysed globally (all segments, all slices), by slice (3 slices), and by LV wall (common wall segments averaged from all 3 slices). HA was also analysed globally, by slice and by wall but also in 3 layers (epicardium, mesocardium, endocardium) because of the known transmural variation in HA. Values from the initial study were compared between LV walls, slices and layers (for HA) using a hierarchical (patients - slices - LV walls - layers) mixed effects model. The lateral wall and mid slice were chosen as reference regions for comparison, because the lateral wall is less commonly affected by hypertrophy in HCM, and the mid-wall is furthest from any anomalous measurement relating to the apex or mitral valve. All values were found to be normally distributed using the Kolmogorov-Smirnov test, and are therefore shown as mean ± standard deviation (SD). Statistical significance was set to p <0.01 to account for the number of statistical tests performed. Interstudy reproducibility was assessed using the SD of the difference between the scans, and the coefficient of variation (CoV) where reasonable (SD of the difference between the scans divided by the measurement mean). Bland-Altman analysis was also performed. Differences in the CoV were assessed using a variance ratio test.

## Results

### Study population

The baseline demographic, clinical and CMR characteristics of the study population are summarised in Table 1.

**Table 1:** **Baseline patient characteristics** [**mean** ± **SD**, **or number of patients** (%)]

	**HCM patients(n** = **10)**
Age [years]	57 ± 9
Male	9 (90%)
Body Surface Area [m^2^]	1.99 ± 0.2
Body mass index [kg/m^2^]	27.5 ± 4.1
**LV morphology &risk factors**	
Asymmetrical septal hypertrophy	7 (70%)
Apical hypertrophy	3 (30%)
Number of risk factors for sudden death: median (range)	1 (0-1)
Presence and location of late gadolinium enhancement	9 (90%)
Septum	47%
Anterior	20%
Inferior	20%
Lateral	7%
Extent of late gadolinium enhancement: [% of LV mass]	4.5% ± 5.6%
**CMR dimensions and function**	
Indexed LV EDV [mL/m^2^]	70 ± 12
Indexed LV ESV [mL/m^2^]	16 ± 4
LV Ejection Fraction [%]	77 ± 5
LV Mass Index [g/m^2^]	63 ± 12
Maximum end-diastolic Wall Thickness [mm]	22 ± 5

### Diffusion tensor imaging

**Figure 1:**
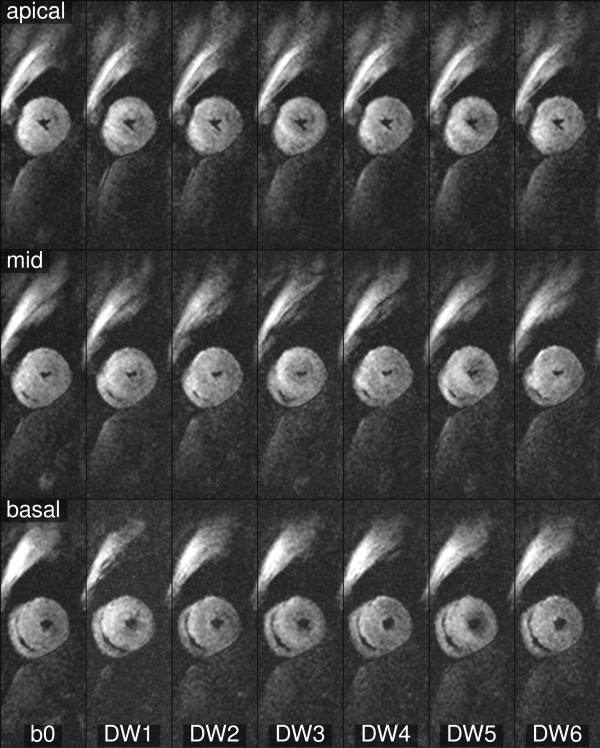
**Example of b0 and diffusion**-**encoded images (one average).** This data demonstrates the typical image quality obtained throughout the study. Multiple data sets were acquired, motion corrected and averaged before cDTI post-processing.

**Figure 2:**
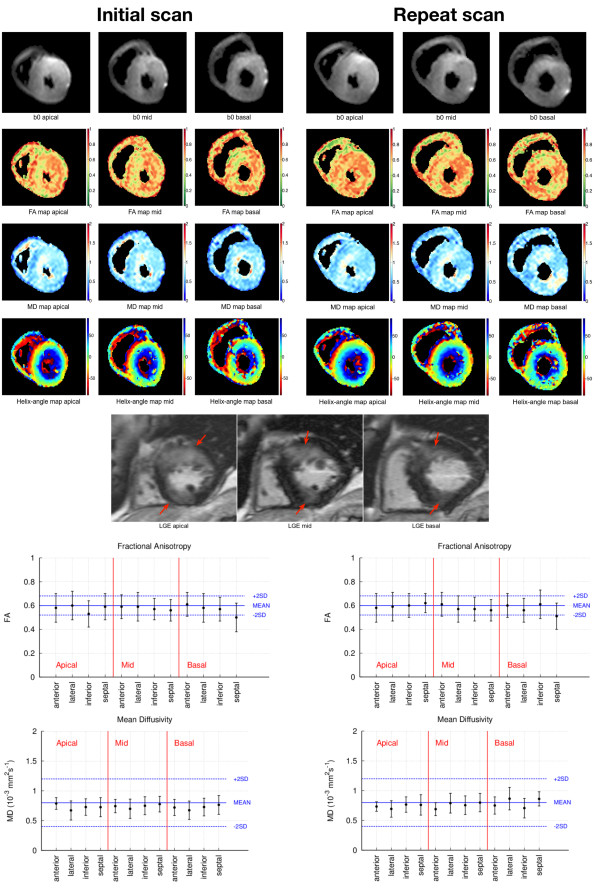
**cDTI data set for initial and repeat scan in a highly reproducible cDTI example.** The three cDTI slices are depicted as b0 images after averaging, with the derived colourised FA, MD and HA maps. The bottom images show the matching LGE slices with arrows highlighting the enhanced regions. The plots show the measured FA and MD values for the myocardial segment regions only (the blue lines represent the healthy mean value and the ± 2SD limits reported by Nielles-Vallespin et al., [22] and the error bars in the data points represent the standard deviation measured in each myocardial segment). No major differences are observed between initial and repeat scans for any parameter map or plots. The HA distribution maps look similar to the ones expected in healthy subjects and do not display obvious disarray in the area of LGE (anterior and inferior) or elsewhere.

**Figure 3:**
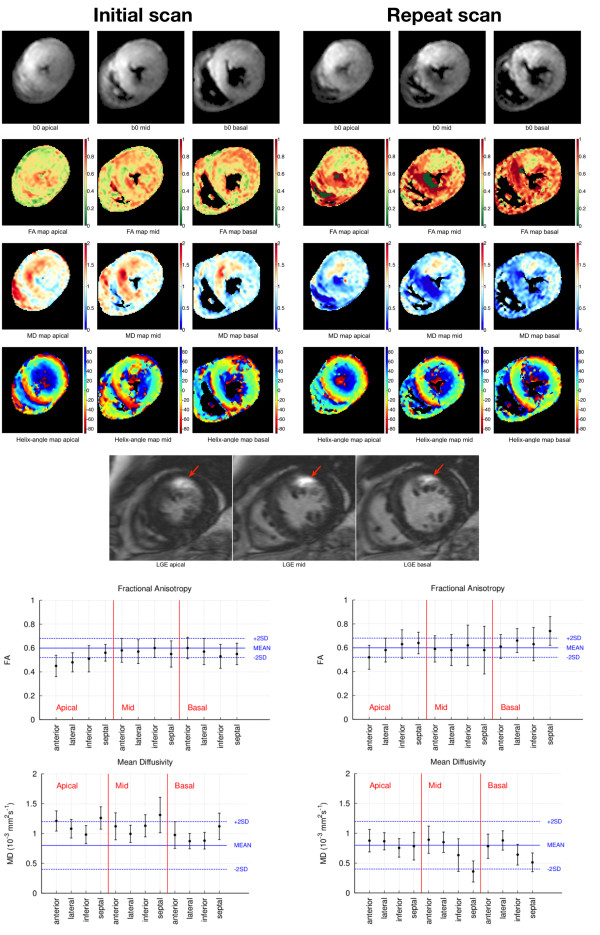
**cDTI data set for initial and repeat scan in the least reproducible example**, **most affected by artefacts data set of the ten investigated in this study.** The three cDTI slices are depicted as b0 images after averaging, with the derived colourised FA, MD and HA maps. The bottom images show the matching LGE slices with arrows highlighting the enhanced regions. The plots show the measured FA and MD values for the myocardial segment regions only (the blue lines represent the healthy mean value and the ± 2SD limits reported by Nielles-Vallespin et al., [22] and the error bars in the data points represent the standard deviation measured in each LV wall). Differences can be seen in FA, MD and HA maps, particularly in the mean diffusivity of the septal region mid-slice due to a signal loss artefact in the b0 images of the repeat scan. The HA distribution maps shows noisy pixels due to motion artefacts which are not reproducible. No definite pattern of change is seen in the area of LGE in the anterior wall or elsewhere.

DTI was successfully acquired in all cases. Figure 1 demonstrates a DTI dataset showing typical images before averaging. b0 images (references images without diffusion weighting) after motion correction and averaging, and derived FA, MD and HA maps at three slice locations, together with FA and MD plots for each LV myocardial segment, both for the initial and repeat scans, are shown in Figures 2 and 3. While Figure 2 displays a reproducible data set without obvious artifacts, Figure 3 displays the least reproducible data set of this study. In Figure 2, the MD and FA maps appear homogeneous, however the transmural progression of HA can be clearly observed and looks as expected in a healthy volunteer. In Figure 3, the HA maps look perturbed.

### Fractional anisotropy

The mean global FA in the 10 patients in the initial study was 0.613 ± 0.044 (Table 2). When analysed by slice, FA was greatest in the mid-slice (0.623 ± 0.043) and smaller, but not significantly so in the basal and apical slices (using the mid slice as reference). When analysed by LV wall, FA was greatest in the anterior wall at 0.615 ± 0.039 and least in the septal wall at 0.610 ± 0.059. Using the lateral wall as a reference, there was no statistically significant difference between walls.

**Table 2:** **Fractional anisotropy** (**FA**): **results from initial study**

**FA**	**N**	**Mean**	**SD**	**Difference**	**95% Confidence interval**		**p value**
**Global**	10	0.613	0.044				
**Slice**							
Mid	10	0.623	0.043	Reference			
Apical	10	0.608	0.05	-0.015	-0.04	0.01	0.25
Basal	10	0.608	0.046	-0.015	-0.04	0.01	0.25
**LV wall**							
Lateral	10	0.614	0.041	Reference			
Anterior	10	0.615	0.039	0.002	-0.021	0.024	0.90
Inferior	10	0.611	0.04	-0.003	-0.026	0.019	0.79
Septal	10	0.61	0.059	-0.003	-0.026	0.019	0.77

### Mean diffusivity

The mean global MD was 0.750 ± 0.154 × 10^-3^ mm^2^/s (Table 3). MD was greatest in the apical slice (0.785 ± 0.152 × 10^-3^ mm^2^/s) and least in the basal slice (0.726 ± 0.145 × 10^-3^ mm^2^/s), but there were no statistically significant differences between slices. When analysed by LV wall, MD was significantly greater in the septal wall than the reference lateral wall (0.784 ± 0.188 × 10^-3^ mm^2^/s vs 0.714 ± 0.155 × 10^-3^ mm^2^/s; p < 0.001).

**Table 3:** **Mean diffusivity** (**MD**): **Results from initial study** [×**10**^-**3**^ **mm**^**2**^/**s**]

**MD**	**N**	**Mean**	**SD**	**Difference**	**95% Confidence interval**		**p value**
**Global**	10	0.75	0.154				
**Slice**							
Mid	10	0.742	0.178	Reference			
Apical	10	0.785	0.152	0.042	-0.017	0.101	0.16
Basal	10	0.726	0.145	-0.016	-0.075	0.043	0.60
**LV wall**							
Lateral	10	0.714	0.155	Reference			
Anterior	10	0.758	0.145	0.044	0.004	0.083	0.029
Inferior	10	0.742	0.137	0.028	-0.011	0.067	0.16
Septal	10	0.784	0.188	0.07	0.031	0.109	<0.001

### Helix angle

Global LV helical angles (Table 4) showed transmural progression from left handed in the epicardium (−34.3 ± 7.6°), near perpendicular to the imaging plane in the mesocardium (3.5 ± 6.9°), to right handed in the endocardium (38.9 ± 8.1°). When analysed by slice, the helical angles were not statistically different between slices. When analysed by LV wall, the endocardial HA was statistically more right handed in the inferior and septal walls compared to the lateral wall (33.9 ± 8.4°, 34.1 ± 5.2° vs 42.6 ± 5.5°; both p < 0.001). In the mesocardial layer the inferior and septal walls HA were significantly more perpendicular to the plane than in the lateral wall (−0.2 ± 5.7°, -0.7 ± 6.9° vs 7.6 ± 4.5°; both p < 0.001). In the epicardial layer, the anterior and septal HA was significantly more left handed than lateral wall (−36.9 ± 6.9°, -39.1 ± 8.2° vs −28.7 ± 6.0° p = 0.004 & <0.001).

**Table 4:** **Helix angle** (**HA**): **Results from initial study** [**degrees**]

**HA**	**N**	**Mean**	**SD**	**Difference**	**95% Confidence interval**		**p value**
**Global**							
Endo	10	38.9	8.1				
Meso	10	3.5	6.9				
Epi	10	−34.3	7.6				
**Slice**							
**Endo layer**							
Mid	10	40	4.6	Reference			
Apical	10	41	7.2	1	−2.6	4.7	0.58
Basal	10	36.3	6	−3.7	−7.4	−0.1	0.044
**Meso layer**							
Mid	10	3	3.2	Reference			
Apical	10	4.3	5.3	1.3	−1.5	4	0.37
Basal	10	3.5	4.3	0.5	−2.2	3.2	0.72
**Epi layer**							
Mid	10	−34.4	3.2	Reference			
Apical	10	−33.8	6.5	0.6	−3	4.2	0.73
Basal	10	−34.8	4.1	−0.4	−4	3.2	0.82
**LV wall**							
**Endo layer**							
Lateral	10	42.6	5.5	Reference			
Anterior	10	45.1	6.6	2.5	−1.6	6.7	0.24
Inferior	10	33.9	8.4	−8.7	−12.8	−4.5	<0.001
Septal	10	34.1	5.2	−8.5	−12.6	−4.3	<0.001
**Meso layer**							
Lateral	10	7.6	4.5	Reference			
Anterior	10	7.3	6.1	−0.4	−4.6	3.9	0.87
Inferior	10	−0.2	5.7	−7.8	−12.1	−3.5	<0.001
Septal	10	−0.7	6.9	−8.3	−12.6	−4	<0.001
**Epi layer**							
Lateral	10	−28.7	6	Reference			
Anterior	10	−36.9	6.9	−8.3	−13.8	−2.7	0.004
Inferior	10	−32.4	5.4	−3.7	−9.3	1.8	0.19
Septal	10	−39.1	8.2	−10.4	−16	−4.9	<0.001

### Reproducibility

**Figure 4:**
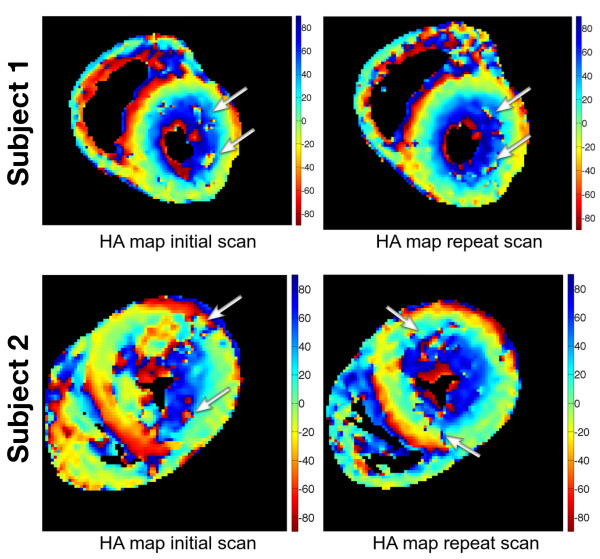
**Two examples of the HA maps measured in the mid slice in the initial and repeat study.** Subject 1 HA maps are very similar between the two studies, while subject 2 HA maps are not as reproducible, particularly in the septal and anterior regions. Small regions with abnormal pixels (arrows) can often be seen in the HA maps. This is not well correlated to regions of LGE and is likely to be related to noise.

Initial and repeat studies were performed 167 ± 21 days apart. There were no clinical events or changes in treatment in the intervening period. FA, MD and HA maps appeared similar between initial and repeat studies in the majority of cases (Figure 2). Figure 4 shows two pairs of HA maps (initial vs repeat study) showing good reproducibility (top), and an example which appears less reproducible (bottom). Small regions with high levels of noise (arrows) can be seen in the HA maps, thought to be a consequence of motion, either cardiac or respiratory or both.

**Figure 5:**
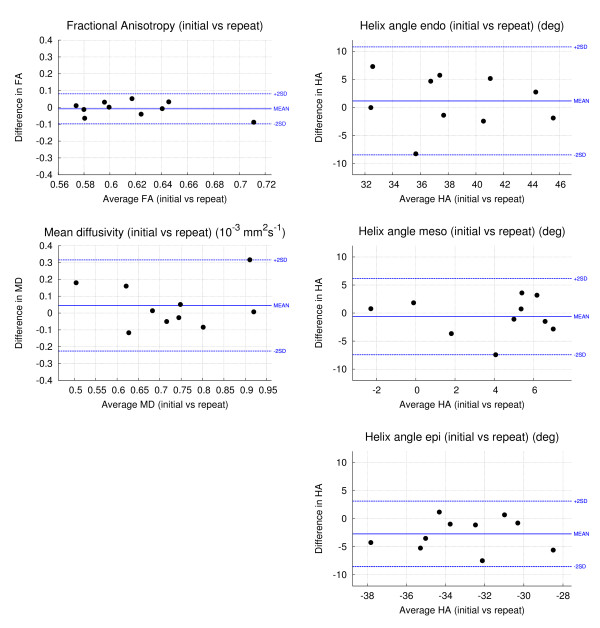
**Bland-Altman plots for the global FA, MD, and HA (endo-, meso-, epicardium) showing the interstudy reproducibility between the initial and repeat studies.**

FA reproducibility data, comparing the initial and repeat studies are provided in Table 5 and the associated Bland-Altman plots are shown in Figure 5. FA data analysed globally, per slice and by LV wall showed good reproducibility with minimal bias and low variance. The global SD between the initial and repeat studies was ± 0.045 and the CoV was 7.2%.

**Table 5:** **Fractional anisotropy** (**FA**) **reproducibility**

**FA**	**N**	**Mean value**	**Mean difference**	**SD of difference**	**95% limits of agreement**		**Coefficient of variation**
**Global**	10	0.617	−0.008	0.045	−0.096	0.079	7.2%
**Slice**							
Apical	10	0.614	−0.013	0.054	−0.119	0.093	8.8%
Mid	10	0.622	0.001	0.051	−0.099	0.102	8.2%
Basal	10	0.614	−0.013	0.043	−0.098	0.072	7.1%
**LV wall**							
Anterior	10	0.620	−0.009	0.042	−0.091	0.073	6.8%
Lateral	10	0.608	0.012	0.059	−0.104	0.128	9.7%
Inferior	10	0.620	−0.018	0.053	−0.123	0.087	8.6%
Septal	10	0.619	−0.017	0.053	−0.121	0.086	8.5%

MD reproducibility data, comparing the initial and repeat studies, are provided in Table 6 and Figure 5. MD data showed good reproducibility at each level of analysis. The global SD between the initial and repeat studies was ± 0.135 × 10^-3^ mm^2^/s and the coefficient of variation was 18.6%. The interstudy reproducibility of MD was significantly less good than for FA (p = 0.003).

**Table 6:** **Mean diffusivity** (**MD**) **reproducibility** (×**10**^-**3**^ **mm**^**2**^/**s**)

**MD**	**N**	**Mean value**	**Mean difference**	**SD of difference**	**95% limits of agreement**		**Coefficient of variation**
**Global**	10	0.728	0.045	0.135	−0.220	0.310	18.6%
**Slice**							
Apical	10	0.764	0.041	0.176	−0.304	0.385	23.0%
Mid	10	0.720	0.045	0.160	−0.268	0.358	22.2%
Basal	10	0.704	0.045	0.115	−0.181	0.271	16.4%
**LV wall**							
Anterior	10	0.727	0.061	0.112	−0.158	0.281	15.4%
Lateral	10	0.713	0.003	0.164	−0.317	0.324	22.9%
Inferior	10	0.716	0.053	0.140	−0.221	0.327	19.5%
Septal	10	0.751	0.067	0.232	−0.388	0.521	30.9%

**Figure 6:**
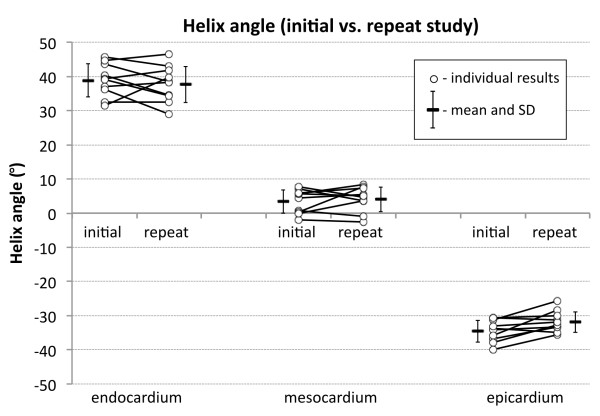
**Line plots of the mean global HA values in the initial and repeat study for the endo**-, **meso**- **and epicardial layers for all 10 subjects.** The mean ± 1SD of each group is also shown.

**Figure 7:**
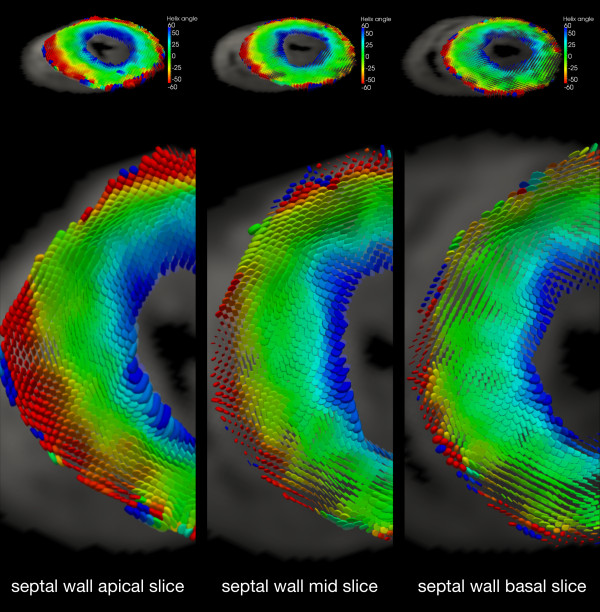
**The diffusion tensor represented by superquadric glyphs in three slices (shown only in the myocardium).** The glyphs represent not only the principal diffusion direction (myocyte orientation) but also the secondary and tertiary eigenvalues along the cross-myocyte directions.

HA reproducibility data, comparing the initial and repeat studies are provided in detail in Table 7. The associated plots are shown in Figures 5 and 6. The global mean SD for HA between the initial and repeat studies was ± 4.8°, ± 3.4° and ± 2.9° in the endocardial, mesocardial and epicardial layers. Analysis of HA by slice and LV wall was similarly reproducible. Three-dimensional analysis of the DTI data is shown in Figure 7 as superquadric glyphs. The colour and orientation/shape of the glyphs are determined by the helix angle and the eigensystem respectively.

**Table 7:** **Helix angle** (**HA**) **reproducibility** (**degrees**)

**HA**	**N**	**Mean value**	**Mean difference**	**SD of difference**	**95% limits of agreement**	
**Global**						
Endo	10	38.4	1.2	4.8	−8.3	10.6
Meso	10	3.9	−0.6	3.4	−7.3	6.0
Epi	10	−33.1	−2.7	2.9	−8.4	3.0
Apical endo	10	39.5	3.0	10.6	−17.8	23.9
Apical meso	10	4.3	0.0	6.1	−12.0	12.0
Apical epi	10	−32.0	−3.6	6.4	−16.2	8.9
Mid endo	10	39.1	1.9	6.2	−10.3	14.1
Mid meso	10	3.4	−0.7	4.7	−9.9	8.4
Mid epi	10	−33.0	−2.9	6.8	−16.2	10.4
Basal endo	10	36.6	−0.8	4.4	−9.3	7.8
Basal meso	10	4.1	−1.1	3.2	−7.3	5.2
Basal epi	10	−33.9	−1.8	4.7	−11.0	7.4
**LV wall**						
Anterior endo	10	43.2	3.7	8.6	−13.2	20.5
Anterior meso	10	6.0	2.6	5.5	−8.1	13.3
Anterior epi	10	−36.5	−0.9	7.3	−15.2	13.5
Lateral endo	10	42.6	0.0	4.9	−9.7	9.7
Lateral meso	10	8.7	−2.2	6.9	−15.8	11.4
Lateral epi	10	−27.7	−2.0	7.8	−17.4	13.3
Inferior endo	10	34.2	−0.6	6.7	−13.6	12.5
Inferior meso	10	0.7	−1.7	7.2	−15.9	12.4
Inferior epi	10	−30.8	−3.2	9.4	−21.7	15.3
Septal endo	10	33.3	1.6	8.0	−14.1	17.4
Septal meso	10	0.2	−1.8	6.8	−15.1	11.6
Septal epi	10	−36.7	−4.9	7.3	−19.3	9.5

The CoV was not calculated for HA, see text for details.

## Discussion

In-vivo cDTI is challenging due to cardiac motion and the intrinsically low SNR of the technique. However, our previous study showed that cDTI using the DW-STEAM sequence at 3T can be performed in-vivo in healthy volunteers with good reproducibility [22]. We now show that this technique can be performed apparently successfully in patients with HCM and have assessed its interstudy reproducibility and the variation of quantitative measures around the myocardium.

FA had a global value of 0.613 ± 0.044 and analysis by slice and wall yielded similar results with no statistical differences. The interstudy reproducibility of global FA was very good at 7.2%, with values ranging from 6.8% to 9.7% for slices and walls. This very robust reproducibility suggests that the values have clinical validity. MD had a global value of 0.750 ± 0.154 × 10^-3^ mm^2^/s and was significantly higher in the septum (0.784 ± 0.188 × 10^-3^ mm^2^/s, p < 0.001). The interstudy reproducibility of global MD was reasonable at 18.6%, with values ranging from 15.4% to 30.9% for slices and walls. The interstudy reproducibility of MD was significantly less good than for FA (p = 0.003).

One possible interpretation of the finding of higher MD in the septum might be that it resulted from the commonly found septal hypertrophy in HCM in association with an enlarged interstitial space from disarray or fibrosis. However, considerable caution must be observed in the interpretation of this result because of known and unknown technical issues. One issue might be that the SNR across the LV is not homogeneous due to the decreasing sensitivity of the receiver surface coils with depth (the signal being higher the closer to the coil elements). It is well documented in diffusion tensor imaging that FA and MD values are biased by SNR due to the Rician noise distribution and due to the fact that only magnitude images are used to derive the diffusion tensor parameters [39]. Therefore, higher SNR may lead to higher values of MD. The evaluation of the SNR in these images is beyond the scope of this paper, but will be the subject of future work. Another possible confounding issue is sensitivity to the difference in myocardial position between the 2 successive cardiac cycles used for imaging, which might affect different regions of the LV in a different manner and create artefactual MD differences across the LV. A further potential issue is mis-registration of the regions of interest used to segment the LV, with influence of right ventricular papillary muscles affecting the septal measurements. Finally, there will also be unidentified technical issues which could have a bearing on the results. The relative contribution of each of these issues is currently unknown, and in-depth analysis of larger numbers of patients and healthy volunteers will be necessary to determine whether the increased MD is an actual clinical finding or caused by limitations of the present cDTI technique.

Analysis of HA was divided into 3 transmural layers because of prior findings. The global value for endocardial HA was 38.9° ± 8.1°, and SD of the difference between the measurement for the 2 scans was 4.8°. For the mesocardium, the global value was 3.5° ± 6.9° with an interstudy SD of 3.4°. For the epicardium, the global value was −34.3° ± 7.6° with an interstudy SD of 2.9°. These findings indicate very good reproducibility between scans of HA in all 3 layers and suggest clinical validity. CoVs were not calculated because their magnitude is a function of the coordinate angles chosen, and in the current system were incalculable in the mesocardial layer as the mean helical angle approximates to zero as myocyte orientation rotates from a left-handed to right-handed helix creating unstable values by division. Significant differences in HA were found between the reference lateral wall and the septum in all 3 transmural layers (ranging from 8.5° to −10.4°, all p < 0.001). Significant differences also affected the inferior wall in the endo and mesocardium (p < 0.001), and the anterior wall in the epicardium (p = 0.004). One possible interpretation of the finding of different HA values in the septum is disturbance from myocardial disarray. However, once again this interpretation needs to be regarded with considerable caution. In this study the myocardium was segmented transmurally into 3 equal thickness layers. This does not necessarily represent the real left-handed, circular and right-handed HA structure of the myocardium and therefore averaging within these 3 layers is bound to happen. Although this should not largely affect the reproducibility, it could influence the LV wall comparison due to the varying myocardial thickness per wall segment. The segmentation might be subject-dependent due to the fact that the papillary muscles and RV wall are visually excluded and might differ between initial and repeat scans, affecting the values due to mis-registration of the region of interest, and also across patients. The design, implementation, optimisation and comparison of segmentation algorithms to extract robust and meaningful quantitative HA data will be a very important task for the translation of cardiac DTI into routine, and is the subject of ongoing research. On the other hand, HA distribution maps appeared healthy in the majority of cases. We hypothesise that this might be due to the fact that the patient cohort scanned for this study was composed of stable HCM patients. There is a possibility that this is the reason why the HA maps appear normal, and that more severe forms of HCM might correlate with disturbed HA distribution maps.

There is relatively little previous in-vivo work on the absolute values of FA and MD. In healthy human subjects we reported an MD value of 0.8 ± 0.2 × 10^-3^ mm^2^/s using a similar breath hold DW-STEAM protocol, [22] and Reese et al. [40] reported an MD of 0.9 ± 0.3 × 10^-3^ mm^2^/s, which are both within one standard deviation of the global value of 0.750 ± 0.154 × 10^-3^ mm^2^/s found in the current study of HCM patients. Tseng et al. performed in-vivo DTI in 5 patients with HCM and 5 volunteers [25]. They measured FA at 0.78 in the free wall and 0.56 in the septum in HCM patients (p = 0.03 for the difference) and 0.78 and 0.72 respectively in normal subjects (p = 0.15 for the difference). In our current study, we measured slightly lower FA at 0.614 ± 0.041 × 10^-3^ mm^2^/s in the lateral wall and 0.610 ± 0.059 × 10^-3^ mm^2^/s in the septum, with no significant difference between our values (p = 0.77). Further studies will be needed to compare measures, standardise and optimise the methods and further validate results. It should be noted that the quantitative results in this study are obtained from acquisitions at end-systole. We might expect differences between in-vivo and ex-vivo data, and also between fresh and fixed ex-vivo data, and finally also between data acquired at different cardiac phases.

The general limitations of this study are the following: The modest in-plane resolution of 2.7 mm might lead to volume averaging of the blood pool in endocardial layers or the RV papillary muscles in epicardial layers, which could bias the FA, MD and HA values in these areas; Data acquisition was performed at end-systole, which is considered a phase when the diffusion acquisition is not affected by strain, [40] and although the systolic pause in HCM is longer than in healthy volunteers, motion artifacts may still have occurred related to the optimal positioning of the acquisition window; The time between the 2 scans in the study might have resulted in alteration in the myocardium due to disease progression, but this is unlikely given the slow rate of progression and the lack of any clinical events in the intervening period; This is a preliminary study on a small sample size of only 10 HCM patients, and further work with a larger number of patients is required, which will include quantitative regional comparisons with analysis of LGE; The application of the current technique to patients with limited breathold capability or frequent ectopy remains to be determined, but would be more challenging.

## Conclusions

In conclusion, we have implemented a robust quantitative in-vivo cDTI technique that has the potential to enhance our understanding of in-vivo structure-function relationships in the HCM heart. We have shown that cDTI of the human HCM heart in-vivo can be performed using a recently reported technique, and we have assessed the reproducibility of the results of this technique in HCM patients.

## Abbreviations

AHA: American Heart Association; cDTI: Cardiac Diffusion Tensor Imaging; CMR: Cardiovascular Magnetic Resonance; CoV: Coefficient of Variation; DTI: Diffusion Tensor Imaging; DW-STEAM: Diffusion Weighted Stimulated Echo Acquisition Mode; EPI: Echo Planar Imaging; FA: Fractional Anisotropy; HA: Helix Angle; LGE: Late Gadolinium Enhancement; LV: Left Ventricle; HCM: Hypertrophic Cardiomyopathy; MD: Mean Diffusivity; SD: Standard Deviation; SNR: Signal to Noise Ratio.

## Competing interests

Prof Dudley Pennell is a consultant to Siemens and a director of Cardiovascular Imaging Solutions. Royal Brompton Hospital has research collaboration agreements with Siemens and Medical Solutions. The other authors declare that they have no competing interests.

## Authors’ contributions

LAM, TFI, SNV, PF, ADS, SKP, DNF recruited patients, acquired patient data and performed image analysis and data handling. MR performed all study statistics. SNV, PF, PS, TF, CM, DES, PDG, ADS, DNF contributed to image acquisition and processing. DJP, DNF, PJK, SYH, KPM, RDS conceived the study. DJP, DNF were responsible for the final manuscript. All authors read and approved the final manuscript.

## Authors’ information

David N Firmin and Dudley J Pennell are joint senior authors.

Laura-Ann McGill, Tevfik F Ismail, Sonia Nielles-Vallespin and Pedro Ferreira are joint first authors.

## References

[B1] Maron BJ Gardin JM Flack JM Gidding SS Kurosaki TT Bild DE Prevalence of hypertrophic cardiomyopathy in a general population of young adults. Echocardiographic analysis of 4111 subjects in the cardia study. Coronary artery risk development in (young) adults Circulation 1995 92 785 9 10.1161/01.CIR.92.4.785 7641357

[B2] Maron BJ Hypertrophic cardiomyopathy: A systematic review JAMA 2002 287 1308 20 10.1001/jama.287.10.1308 11886323

[B3] Elliott P McKenna WJ Hypertrophic cardiomyopathy Lancet 2004 363 1881 91 10.1016/S0140-6736(04)16358-7 15183628

[B4] Hughes SE The pathology of hypertrophic cardiomyopathy Histopathology 2004 44 412 27 10.1111/j.1365-2559.2004.01835.x 15139989

[B5] Maron BJ Anan TJ Roberts WC Quantitative analysis of the distribution of cardiac muscle cell disorganization in the left ventricular wall of patients with hypertrophic cardiomyopathy Circulation 1981 63 882 94 10.1161/01.CIR.63.4.882 7193536

[B6] Maron BJ Roberts WC Hypertrophic cardiomyopathy and cardiac muscle cell disorganization revisited: Relation between the two and significance Am Heart J 1981 102 95 110 10.1016/0002-8703(81)90419-1 7195645

[B7] Kuribayashi T Roberts WC Myocardial disarray at junction of ventricular septum and left and right ventricular free walls in hypertrophic cardiomyopathy Am J Cardiol 1992 70 1333 40 10.1016/0002-9149(92)90771-P 1442587

[B8] Maron BJ Wolfson JK Roberts WC Relation between extent of cardiac muscle cell disorganization and left ventricular wall thickness in hypertrophic cardiomyopathy Am J Cardiol 1992 70 785 90 10.1016/0002-9149(92)90560-L 1519531

[B9] Becker AE Caruso G Myocardial disarray. A critical review Br Heart J 1982 47 527 38 10.1136/hrt.47.6.527 7044398 PMC481178

[B10] Bulkley BH D'Amico B Taylor AL Extensive myocardial fiber disarray in aortic and pulmonary atresia. Relevance to hypertrophic cardiomyopathy Circulation 1983 67 191 8 10.1161/01.CIR.67.1.191 6681520

[B11] St John Sutton MG Lie JT Anderson KR O'Brien PC Frye RL Histopathological specificity of hypertrophic obstructive cardiomyopathy. Myocardial fibre disarray and myocardial fibrosis Br Heart J 1980 44 433 43 10.1136/hrt.44.4.433 7191711 PMC482424

[B12] Gersh BJ Maron BJ Bonow RO Dearani JA Fifer MA Link MS Naidu SS Nishimura RA Ommen SR Rakowski H Seidman CE Towbin JA Udelson JE Yancy CW 2011 accf/aha guideline for the diagnosis and treatment of hypertrophic cardiomyopathy: A report of the American college of cardiology foundation/American heart association task force on practice guidelines. Developed in collaboration with the American association for thoracic surgery, American society of echocardiography, American society of nuclear cardiology, heart failure society of America, heart rhythm society, society for cardiovascular angiography and interventions, and society of thoracic surgeons J Am Coll Cardiol 2011 58 e212 60 10.1016/j.jacc.2011.06.011 22075469

[B13] Maron MS Clinical utility of cardiovascular magnetic resonance in hypertrophic cardiomyopathy J Cardiovasc Magn Reson 2012 14 13 10.1186/1532-429X-14-13 22296938 PMC3293092

[B14] Noureldin RA Liu S Nacif MS Judge DP Halushka MK Abraham TP Ho C Bluemke DA The diagnosis of hypertrophic cardiomyopathy by cardiovascular magnetic resonance J Cardiovasc Magn Reson 2012 14 17 10.1186/1532-429X-14-17 22348519 PMC3309929

[B15] Rickers C Wilke NM Jerosch-Herold M Casey SA Panse P Panse N Weil J Zenovich AG Maron BJ Utility of cardiac magnetic resonance imaging in the diagnosis of hypertrophic cardiomyopathy Circulation 2005 112 855 61 10.1161/CIRCULATIONAHA.104.507723 16087809

[B16] Ismail TF Prasad SK Pennell DJ Prognostic importance of late gadolinium enhancement cardiovascular magnetic resonance in cardiomyopathy Heart 2012 98 438 42 10.1136/heartjnl-2011-300814 22128204

[B17] O'Hanlon R Grasso A Roughton M Moon JC Clark S Wage R Webb J Kulkarni M Dawson D Sulaibeekh L Chandrasekaran B Bucciarelli-Ducci C Pasquale F Cowie MR McKenna WJ Sheppard MN Elliott PM Pennell DJ Prasad SK Prognostic significance of myocardial fibrosis in hypertrophic cardiomyopathy J Am Coll Cardiol 2010 56 867 74 10.1016/j.jacc.2010.05.010 20688032

[B18] Petersen SE Jerosch-Herold M Hudsmith LE Robson MD Francis JM Doll HA Selvanayagam JB Neubauer S Watkins H Evidence for microvascular dysfunction in hypertrophic cardiomyopathy: New insights from multiparametric magnetic resonance imaging Circulation 2007 115 2418 25 10.1161/CIRCULATIONAHA.106.657023 17452610

[B19] Sosnovik DE Wang R Dai G Reese TG Wedeen VJ Diffusion MR tractography of the heart J Cardiovasc Magn Reson 2009 11 47 10.1186/1532-429X-11-47 19912654 PMC2781805

[B20] Le Bihan D Looking into the functional architecture of the brain with diffusion MRI Nat Rev Neurosci 2003 4 469 80 10.1038/nrn1119 12778119

[B21] Edelman RR Gaa J Wedeen VJ Loh E Hare JM Prasad P Li W In vivo measurement of water diffusion in the human heart Magn Reson Med 1994 32 423 8 10.1002/mrm.1910320320 7984077

[B22] Nielles-Vallespin S Mekkaoui C Gatehouse P Reese TG Keegan J Ferreira PF Collins S Speier P Feiweier T de Silva R Jackowski MP Pennell DJ Sosnovik DE Firmin D In vivo diffusion tensor MRI of the human heart: Reproducibility of breath-hold and navigator-based approaches Magn Reson Med 2012 Sep 21. [Epub ahead of print] 10.1002/mrm.24488 PMC3864770 23001828

[B23] Riplinger CM Li W Hadley J Chen J Rothenberg F Lombardi R Wickline SA Marian AJ Efimov IR Enhanced transmural fiber rotation and Connexin 43 heterogeneity are associated with an increased upper limit of vulnerability in a transgenic rabbit model of human hypertrophic cardiomyopathy Circ Res 2007 101 1049 57 10.1161/CIRCRESAHA.107.161240 17885214 PMC2366809

[B24] Lombardi R Rodriguez G Chen SN Ripplinger CM Li W Chen J Willerson JT Betocchi S Wickline SA Efimov IR Marian AJ Resolution of established cardiac hypertrophy and fibrosis and prevention of systolic dysfunction in a transgenic rabbit model of human cardiomyopathy through Thiol-sensitive mechanisms Circulation 2009 119 1398 1407 10.1161/CIRCULATIONAHA.108.790501 19255346 PMC2773801

[B25] Tseng WY Dou J Reese TG Wedeen VJ Imaging myocardial fiber disarray and intramural strain hypokinesis in hypertrophic cardiomyopathy with MRI J Magn Reson Imaging 2006 23 1 8 10.1002/jmri.20473 16331592

[B26] Feinberg DA Hoenninger JC Crooks LE Kaufman L Watts JC Ara-kawa M Inner volume MR imaging: technical concepts and their application Radiology 1985 156 743 47 4023236 10.1148/radiology.156.3.4023236

[B27] Griswold MA Jakob PM Heidemann RM Nittka M Jellus V Wang J Kiefer B Haase A Generalized autocalibrating partially parallel acquisitions (GRAPPA) Magn Reson Med 2002 47 1202 10 10.1002/mrm.10171 12111967

[B28] Kramer CM Barkhausen J Flamm SD Kim RJ Nagel E Standardized cardiovascular magnetic resonance imaging (CMR) protocols, society for cardiovascular magnetic resonance: board of trustees task force on standardized protocols J Cardiovasc Magn Reson 2008 10 35 10.1186/1532-429X-10-35 18605997 PMC2467420

[B29] Guizar-Sicairos M Thurman ST Fienup JR Efficient subpixel image registration algorithms Opt Lett 2008 33 156 58 10.1364/OL.33.000156 18197224

[B30] Kingsley PB Introduction to diffusion tensor imaging mathematics: part III. Tensor calculation, noise, simulations, and optimization Concepts Magn Reson 2006 28A 155 79 10.1002/cmr.a.20050

[B31] Basser PJ Inferring microstructural features and the physiological state of tissues from diffusion-weighted images NMR Biomed 1995 8 333 44 10.1002/nbm.1940080707 8739270

[B32] Wu EX Wu Y Nicholls JM Wang J Liao S Zhu S Lau CP Tse HF Mr diffusion tensor imaging study of postinfarct myocardium structural remodeling in a porcine model Magn Reson Med 2007 58 687 95 10.1002/mrm.21350 17899595

[B33] Streeter DD Jr Hanna WT Engineering mechanics for successive states in canine left ventricular myocardium. II. Fiber angle and sarcomere length Circ Res 1973 33 656 64 10.1161/01.RES.33.6.656 4762007

[B34] Streeter DD Jr Hanna WT Engineering mechanics for successive states in canine left ventricular myocardium. I. Cavity and wall geometry Circ Res 1973 33 639 55 10.1161/01.RES.33.6.639 4762006

[B35] Cerqueira MD Weissman NJ Dilsizian V Jacobs AK Kaul S Laskey WK Pennell DJ Rumberger JA Ryan T Verani MS Standardized myocardial segmentation and nomenclature for tomographic imaging of the heart: a statement for healthcare professionals from the cardiac imaging committee of the council on clinical cardiology of the American heart association Circulation 2002 105 539 42 10.1161/hc0402.102975 11815441

[B36] Schultz T Kindlmann GL Superquadric glyphs for symmetric second-order tensors IEEE Trans Vis Comput Graph 2010 16 1595 1604 20975202 10.1109/TVCG.2010.199

[B37] Maceira AM Prasad SK Khan M Pennell DJ Reference right ventricular systolic and diastolic function normalized to age, gender and body surface area from steady-state free precession cardiovascular magnetic resonance Eur Heart J 2006 27 2879 88 10.1093/eurheartj/ehl336 17088316

[B38] Maceira AM Prasad SK Khan M Pennell DJ Normalized left ventricular systolic and diastolic function by steady state free precession cardiovascular magnetic resonance J Cardiovasc Magn Reson 2006 8 417 26 10.1080/10976640600572889 16755827

[B39] Jones DK Basser PJ "Squashing peanuts and smashing pumpkins": how noise distorts diffusion-weighted MR data Magn Reson Med 2004 52 979 93 10.1002/mrm.20283 15508154

[B40] Reese TG Weisskoff RM Smith RN Rosen BR Dinsmore RE Wedeen VJ Imaging myocardial fiber architecture in vivo with magnetic resonance Magn Reson Med 1995 34 786 91 10.1002/mrm.1910340603 8598805

