# Correction: reproducibility of in-vivo diffusion tensor cardiovascular magnetic resonance in hypertrophic cardiomyopathy

**DOI:** 10.1186/1532-429X-15-22

**Published:** 2013-02-28

**Authors:** Laura-Ann McGill, Tevfik Ismail, Sonia Nielles-Vallespin, Pedro Ferreira, Andrew D Scott, Michael Roughton, Philip J Kilner, S Yen Ho, Karen P McCarthy, Peter D Gatehouse, Ranil de Silva, Peter Speier, Thorsten Feiweier, Choukkri Mekkaoui, David E Sosnovik, Sanjay K Prasad, David N Firmin, Dudley J Pennell

**Affiliations:** 1 Cardiovascular Magnetic Resonance Unit, Royal Brompton Hospital, London, UK; 2 National Heart and Lung Institute, Imperial College, London, UK; 3 National Heart Lung and Blood Institute (NHLBI), National Institutes of Health (NIH), DHHS, Bethesda, MD, USA; 4 MR R&D, Siemens AG Medical Solutions, Erlangen, Germany; 5 Martinos Center for Biomedical Imaging, Massachusetts General Hospital, Harvard Medical School, Boston, MA, USA

## 

Following publication of article [1] we noted an error in the methods section of our abstract in which we state: “Global MD was significantly higher in the septum than the reference lateral wall (0.784 ± 0.188 vs 0.750 ± 0.154 × 10^-3^ mm^2^/s, p < 0.001).” The word “Global” was not correct, and the reference value was incorrect, this should state: “MD was significantly higher in the septum than the reference lateral wall (0.784 ±0.188 vs 0.714 ±0.155 × 10^-3^ mm^2^/s, p <0.001).” All other information is correct, we apologize for any inconvenience caused by this error.
